# Eddy, drift wave and zonal flow dynamics in a linear magnetized plasma

**DOI:** 10.1038/srep33371

**Published:** 2016-09-15

**Authors:** H. Arakawa, S. Inagaki, M. Sasaki, Y. Kosuga, T. Kobayashi, N. Kasuya, Y. Nagashima, T. Yamada, M. Lesur, A. Fujisawa, K. Itoh, S.-I. Itoh

**Affiliations:** 1 Teikyo University, 6-22 Misakimachi, Omuta-city, Fukuoka 836-8505, Japan; 2 Research Institute for Applied Mechanics, Kyushu University, 6-1 Kasuga-Koen, Kasuga-city, Fukuoka 816-8580, Japan; 3 Research Center for Plasma Turbulence, Kyushu University, 6-1 Kasuga-Koen, Kasuga-city, Fukuoka 816-8580, Japan; 4 National Institute for Fusion Science, National Institutes of Natural Sciences, 322-6 Oroshi-cho, Toki-city, Gifu 509-5292, Japan; 5 Faculty of Arts and Science, Kyushu University, 744 Motooka, Nishi-ku, Fukuoka 819-0395, Japan; 6 Lorraine University, 54506 Vandoeuvre-lès-Nancy, France

## Abstract

Turbulence and its structure formation are universal in neutral fluids and in plasmas. Turbulence annihilates global structures but can organize flows and eddies. The mutual-interactions between flow and the eddy give basic insights into the understanding of non-equilibrium and nonlinear interaction by turbulence. In fusion plasma, clarifying structure formation by Drift-wave turbulence, driven by density gradients in magnetized plasma, is an important issue. Here, a new mutual-interaction among eddy, drift wave and flow in magnetized plasma is discovered. A two-dimensional solitary eddy, which is a perturbation with circumnavigating motion localized radially and azimuthally, is transiently organized in a drift wave – zonal flow (azimuthally symmetric band-like shear flows) system. The excitation of the eddy is synchronized with zonal perturbation. The organization of the eddy has substantial impact on the acceleration of zonal flow.

Turbulence can play dominant roles in flow structure formation in various plasmas, *e*.*g*., the sun, geospherical plasma, astronomical plasma and fusion plasma^1^,^2^,^3^,^4^,^5^,^6^. The drift wave turbulence excited by the density gradients in magnetized plasmas has many features in common with Rossby waves turbulence in geophysical fluids^7^,^8^. Once excited, drift/Rossby wave turbulence excite zonal flows, which are azimuthally symmetric band-like shear flows^7^,^9^. The zonal flows suppress the turbulence and this nonlinear interaction has been demonstrated by experiments^10^. Formation of zonal flows is critical for fusion plasmas as they can improve confinement^7^,^11^. Thus clarifying its formation mechanism is an important issue. While collisions play a role in determining overall flow level^7^,^12^, nonlinear interaction dominates in collisionless plasmas. Many mechanisms, *e*.*g*., higher order kinetics of flow-fluctuation interaction^13^,^14^, possible role of eddies in phase space^15^, interaction with Generalized Kelvin-Helmholtz (GKH)^16^ etc., have been discussed theoretically. In addition, in planetary and geophysical fluids, such as the Jovian atmosphere, eddies of various scales are also organized in zonal flow-Rossby wave dynamics^17^. This indicates the importance of studying nonlinear interactions in the system of drift wave, zonal flows and eddies. However, how eddies are formed in plasmas and how they interact with turbulence and flows have not been fully clarified in plasma physics. Especially, the previous experimental studies considered that the drift wave *directly* generates and interacts with zonal flows^10^, *neglecting the effect of intermittently generated and spatially localized structure (i.e. eddies).*

Here we report the discovery of the solitary eddy, *intermittently* organized 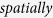 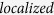 structure, which works as 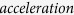 of zonal flow in a cylindrical magnetized plasma. Coexistence of flow, turbulence and eddy, and interactions among them, are studied experimentally. Spatio-temporal correlations among the solitary eddy, drift waves and zonal flow are observed, and the energy partition among them is analyzed. The eddy intermittently appears around an inner antinode of the zonal flow, during a acceleration phase of zonal flow (which is toward the electron diamagnetic direction). The total kinetic energy of eddy is the same order of magnitude of that of zonal flows. A new channel of momentum transfer from the drift wave to zonal flow via solitary eddy is found by evaluating Reynolds stress. The azimuthal force associated with the eddy, the drift waves, and the zonal flow, all have the same order of magnitude, where the azimuthal force is averaged on the same radius. The organization of the eddy has substantial impact on the acceleration of zonal flows.

## Results

### Experimental condition

The experiments were performed on the Large Mirror Device Upgrade (LMD-U)^18^. A cylindrical plasma with a diameter of approximately 0.1 m and an axial length of 3.74 m is radially confined by the axial magnetic field (*z*: axial, *r*: radial, *θ*: azimuthal direction). The operation conditions are 3 kW RF power, 900 G magnetic field and 5 mTorr argon gas pressure with no external sources of momentum input. In this experimental condition, the dominant drive of the azimuthal flow is the azimuthal Reynolds stress^19^,^20^. The major diagnostic tools are a 64-channel azimuthal Langmuir probe array^21^,^22^,^23^ and a radially movable Langmuir probe^18^. The axial and radial positions of the 64-channel probe array in the LMD-U are *z* = 1.885 m and *r *= 4 cm, respectively. In this probe array, each probe measures the ion saturation current, *I*_is_. The radially movable probe is installed at the axial position 1.375 m and *r* = 2–6 cm. The movable probe has three collinear tips; the central tip measures the ion saturation current, *I*_is_ and the other tips measure the floating potential, *V*_f,up/down_. The three tips are separated by 5 mm. These diagnostic tools are operated simultaneously during the discharge.

### Observations of the solitary eddy in nonlinear drift wave – zonal flow system

**Figure 1:**
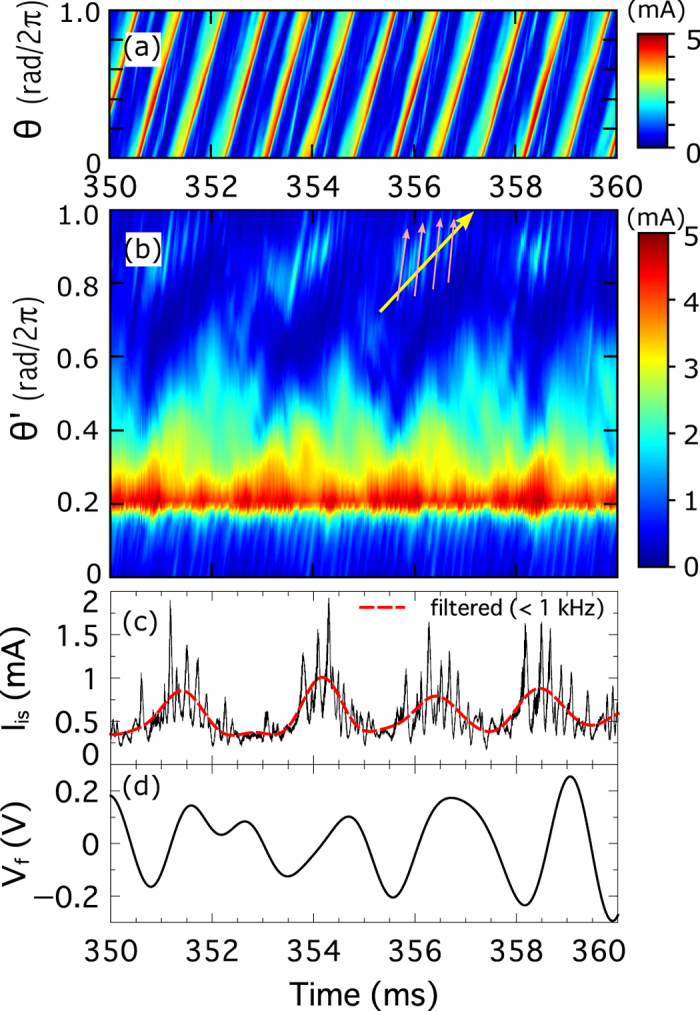
(**a**) Spatio-temporal evolutions of ion saturation current (*I*_is_) measured by the 64-channel azimuthal probe arrays at *r* = 4 cm. (**b**) Temporal evolutions of reconstructed azimuthal structures of nonlinear drift wave by the azimuthal probe arrays. The azimuthal angle (*θ*) of the vertical axis in (**a**) is transformed to *θ*′ = *θ* − *v*_p_*t*, where *v*_p_ indicates the phase velocity of the nonlinear drift wave. The pink and yellow arrows indicate the ‘splash’ and ‘density bump of solitary eddy’. (**c**) Time evolutions of *I*_is_ at (*θ* = 0.94 rad/2*π* (**a**) (solid black line) and low passed (<1 kHz) data (dotted red line). (**d**) Floating potential fluctuation (0.1–1 kHz) at *r* = 4 cm measured by the movable probe.

Figure 1(a) shows the spatio-temporal evolution of *I*_is_ measured by an azimuthal probe array (*r* = 4 cm). A wave coherently propagates in the electron diamagnetic direction. The duration of the propagation is much longer than the period of the drift wave. This wave is called as a nonlinear drift wave. The mechanism to form triangular wave form of drift wave was explained in ref. 24. Although the nonlinear drift wave is stationary, its wave form slightly varies as it propagates. To observe details of temporal changes of azimuthal structure of the nonlinear drift wave, we reconstructed *I*_is_ in the ‘mode frame’ of the nonlinear drift wave, as shown in Fig. 1(b). Here, the azimuthal phase velocity of nonlinear drift wave was subtracted; the azimuthal angle *θ* is transformed to *θ*′ = *θ* − *v*_p_*t*, where *v*_p_ indicates the phase velocity of the nonlinear drift wave (2.4 × 10^3^ *π* rad/s). The azimuthal structure of the nonlinear drift wave shows clear variation. In addition, a small sub-structure is found around *θ*′ = 0.7–1 rad/2*π* in Fig. 1(b). The sub-structure consists of two components with different phase velocities (or frequencies) in laboratory frame (*i*.*e*., including *E* × *B* velocity). One propagates at 4 × 10^3^ *π* rad/s (pink colored arrows on Fig. 1(b)) and this component is called as ‘splash’ (*I*_is,SP_)^25^. The splash has higher frequency than that of the fundamental drift waves and hence has a short life time. The other propagates at the velocity of 2.6 × 10^3^ *π* rad/s (yellow colored arrow on Fig. 1(b)) and this component is density bump associated with ‘solitary eddy’ (discussed later). Figure 1(c) shows the temporal evolution of the sub-structure (slice at *θ*′ = 0.94/2*π* rad of Fig. 1(b)). To separate the splash and the density bump associated with solitary eddy from the sub-structure, high-pass (>1 kHz) and low-pass (<1 kHz) filters were applied to the signal in Fig. 1(c). The splash and the density bump were amplitude-modulated at ~0.4 kHz simultaneously. The temporal evolution of the floating potential fluctuation (0.1–1 kHz) at r = 4 cm of movable probe is shown in Fig. 1(d). A previous work indicated that the excitation of splash is synchronized with the periodic evolution of the zonal perturbation (~0.4 kHz)^25^. In this experiment, in addition to splash, the solitary eddy is observed to form and to interact with zonal perturbation.

### Cross-section image of the solitary eddy and interactions with drift wave and zonal flow

**Figure 2:**
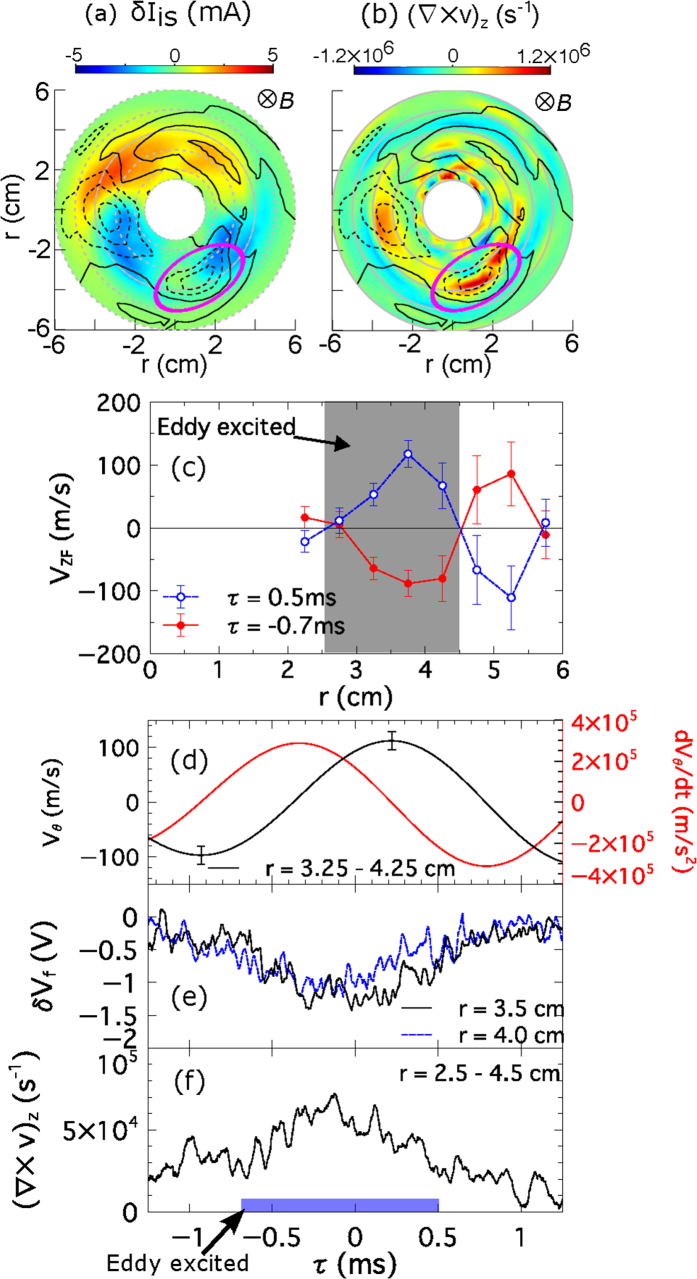
Two-dimensional filled contour structure of (**a**) the ion saturation fluctuation (*δI*_is_) and (**b**) the vorticity (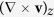) at *τ* = 0. The contour lines indicate the floating potential fluctuation (solid line or dotted line indicate positive or negative value). (**c**) Radial location of the solitary eddy (hatched region) overlapped with the azimuthal zonal flow (*V*_ZF_) at *τ* = −0.7 ms and 0.5 ms. (**d**) Time evolution of zonal perturbation (Black line) and its acceleration (Red line). Positive *V*_ZF_ indicates the electron diamagnetic direction. (**e**) Potential fluctuation at the azimuthal position of the solitary eddy (at *r* = 3.5 and 4 cm). (**f**) Vorticity, averaged radially (*r* = 2.5–4.5 cm) and azimuthally at the solitary eddy location.

Figure 2(a) shows the reconstructed two-dimensional structure of *δI*_is_ and δ*V*_f_ at *τ* = 0 (see ‘Methods’), where filled contours denote *δI*_is_ and contour lines denote *δV*_f_ (solid line or dotted line indicates positive or negative value). Behind the nonlinear drift wave, we can see a closed isoline of *δV*_f_, which is emphasized by a purple solid circle. This indicates the organization of a solitary (radially and azimuthally localized) eddy, since isolines of potential is equivalent to lines of flow in magnetized plasmas. The time-to-peak of the potential perturbation (solitary eddy) was shifted from that of the density bump. Figure 2(b) shows the reconstructed two-dimensional structure of the vorticity, 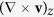, and *δV*_f_ at *τ* = 0, where filled contours denote 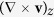 and contour lines denote *δV*_f_. The vorticity peak in the purple solid circle also indicates the organization of the solitary eddy.

The spatio-temporal structure of the solitary eddy is closely related to that of the zonal flow as shown in Fig. 2(c–f). The organized solitary eddy shows spatial and temporal asymmetry with zonal flow. The temporal behavior of azimuthal zonal flow, *V*_ZF_, is evaluated from the radial electric field with 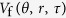. The evaluated *V*_ZF_ structure clearly demonstrates the formation of zonal flow in the region *r *= 2–6 cm as shown in Fig. 2(c). The positive/negative sign of *V*_ZF_ denotes the electron/ion diamagnetic direction. Here, the azimuthal mean flow velocity is ~2.5 km/s at *r *= 3.75 cm in the electron diamagnetic direction. The solitary eddy is organized around an inner antinode of the zonal flow (hatched region of 2.5–4.5 cm). No eddy is observed in the outer antinode of the zonal flow. In this sense, the generation of eddy has a symmetry breaking with respect to the phase of zonal flow. Therefore, the eddy is not excited by the zonal flow shear. Note that the maximum velocity around the solitary eddy is ~1 km/s and is much faster than zonal flow (~0.1 km/s). Figure 2(d) indicates that the zonal flow at *r* = 3.75 cm changes its direction at *τ *= −0.4 ms. Figure 2(e) shows the temporal evolution of *δV*_f_ at *r *= 3.5 cm and 4 cm on an azimuthal position where the solitary eddy is located. Figure 2(f) shows the temporal evolution of vorticity, 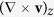, averaged around the solitary eddy location. The averaged vorticity during a cycle of zonal flow is −1 × 10^4^ *s*^−1^. Variations in vorticity are synchronized with variations in zonal flow. The solitary eddy lives at *τ* = −0.7–0.5 ms. The vorticity peaks around 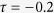 ms. The eddy lives during a phase where the azimuthal flow is accelerated toward the electron diamagnetic direction.

**Figure 3:**
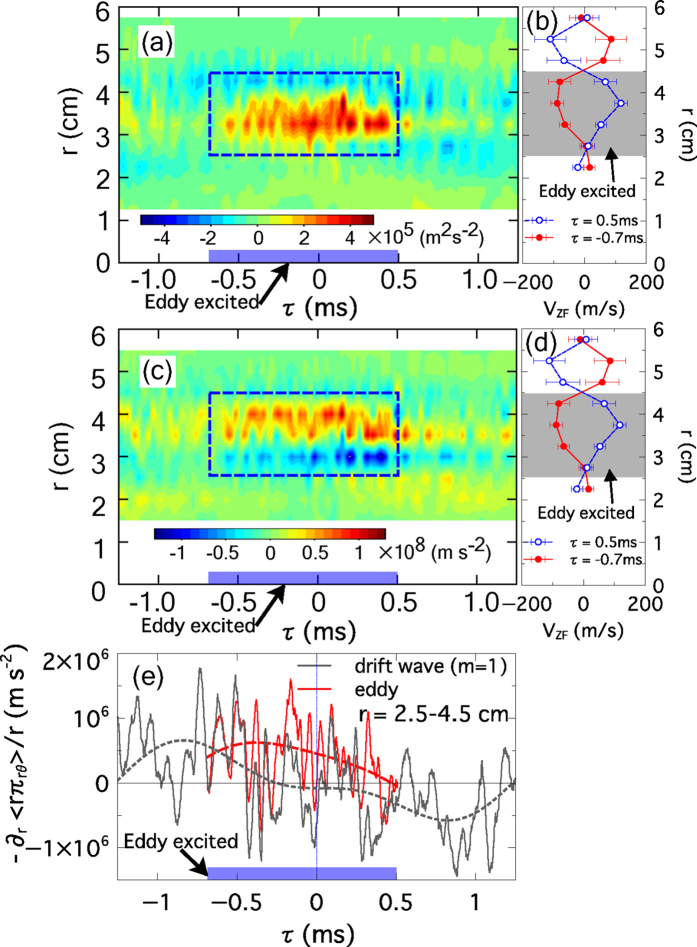
(**a**) Temporal behavior of Reynolds stress, 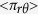, volume-averaged around the azimuthal position of solitary eddy. The dotted square indicates the excited time and radius of the solitary eddy. (**b,d**) Radial profile of zonal flow at 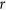 = −0.7 and 0.5 ms (same as Fig. 2(c)). (**c**) Temporal behavior of Reynolds force, 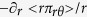. The dotted square indicates the excited time and radius of the solitary eddy, respectively. (**e**) Temporal behavior of Reynolds force around the eddy (−0.7 < *τ* < 0.5 ms) and drift wave (*m* = 1) volume-averaged over *r* = 2.5–4.5 cm. Dotted lines indicate the frequency filtered (0.1–1 kHz) data.

The solitary eddy is organized quasi-periodically, synchronized with zonal flow, and it strengthens the electron diamagnetic flow. Figure 3(a) shows the temporal behavior of the Reynolds stress per mass density, volume-averaged around the azimuthal location of the solitary eddy, which is evaluated using 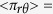

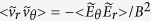
. The dotted square indicates the excited time and radius of the solitary eddy (*τ* = −0.7 ms–0.5 ms, 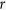 = 2.5–4.5 cm). The Reynolds stress is generated at the radial location where the eddy and zonal flow exist as shown in Fig. 3(b). The Reynolds force, which accelerates/decelerates azimuthal flow is calculated from the radial gradient of Reynolds stress, 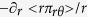. The temporal behavior of Reynolds force is shown in Fig. 3(c), where a positive sign indicates the electron diamagnetic direction. Figure 3(e) shows the fluctuation component of Reynolds force associated with eddy (−0.7 < τ < 0.5 ms) and drift wave (*m* = 1) volume-averaged over the radial location of the eddy (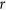 = 2.5–4.5 cm). Here, the solid red line denotes the eddy-driven component and dotted line means the bandpass filtered (0.1–1 kHz) data. The black lines indicate the Reynolds force driven by drift wave (*m* = 1). Although the noise components are large, the filtered data indicates that the eddy accelerates the azimuthal flow toward electron diamagnetic direction which is synchronized with acceleration of the zonal flow as shown in Fig. 2(d). The drift wave also contributes to azimuthal flow which accelerates toward electron (or ion) diamagnetic direction at *τ* < 0 ms (or *τ* > 0 ms).

The effect of the solitary eddy on the zonal flow is substantially large. The dynamic behavior of zonal flow, explained above, cannot be explained by the Reynolds force driven by the drift wave. The phase of acceleration of the zonal flow is delayed from the Reynolds force driven by the drift wave as shown in Figs 2(d) and 3(e). In addition, the magnitude of Reynolds force driven by the eddy has the same order as that driven by the drift wave. Therefore, Reynolds force driven by the solitary eddy can, not only accelerates flow by the same order of magnitude as drift waves, but also governs the dynamics of zonal flow acceleration, as shown by the time-to-peak comparison in Fig. 3(e). The solitary eddy thus plays an important role on excitation and saturation process of zonal flow.

The energy balance among solitary eddy, zonal flow and drift wave is evaluated. For each entity, the energy per unit volume is calculated from squared flow velocity evaluated by floating potential. The energy density of solitary eddy and drift wave are ~10^−2 ^J*m*^−3^; the energy of zonal flow is one order of magnitude lower than these of solitary eddy and drift wave. Taking into account that the eddy has small volume, the energy partition among drift wave, solitary eddy and zonal flow is approximately 10:1:1.

## Discussion

Our result suggests a new channel of energy and momentum transfer from the drift wave to zonal flow via solitary eddy. In this channel, the energy density of the eddy is 1/10 of that of drift wave but the eddy efficiently transfer its momentum to the zonal flow and thus momentum transferred to the zonal flow from the eddy is the same as that from drift wave. Thus, the role of eddy on the momentum transport is significant. The experimental discovery of this new channel indicates the need for a novel picture of plasma turbulence, in which wave, flow and eddy coexist and interact with each other. The role of eddies in drift wave – zonal flow system has also been discussed theoretically^26^. The result presented here will open the way to enhance the zonal flow in fusion plasmas. This new picture is also beneficial in research of the non-equilibrium non-linear systems.

## Methods

### Large Mirror Device-Upgrade (LMD-U)

The LMD-U^18^ is a linear magnetized plasma device; The cylindrical plasma with the diameter of approximately 0.1 m and the axial length of 3.74 m is produced by the RF wave in a quartz tube and is radially bounded by the magnetic field. The vacuum chamber of the LMD-U is made of the stainless steel with the diameter of 0.445 m and the axial length of 3.74 m. Neutral gas (argon) is filled from the plasma source. The ionized vacuum indicator is mounted on the source to monitor the pressure of neutral gas. The mass flow controller controls the amount of neutral gas flow. Four turbo-molecular pumps are installed in nearly middle and end regions of the LMD-U. The total exhaust velocity is 1000 l/s. Those pumps evacuate the background neutral pressure (< 10^−3^ Pa in this study) and reduce impurity gases (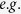 N_2_ and H_2_O gases in the atmosphere). The baffle plates with the inner diameter of 150 mm are installed at the source and end region to stabilize the gas flow and to improve the control. The coil system in the LMD-U produces a linear magnetic field configuration. Strength of the magnetic field is controlled by the coil current. The coil current is excited stationary during the discharge. The LMD-U plasma is produced by helicon wave heating. The helicon wave (*m* = 0, *m*: azimuthal mode number) at 7 MHz is excited with a double loop antenna made of copper, therefore, the plasma does not have external azimuthal momentum sources. The heating power of 3 kW is supplied to the antenna through a matching circuit. The matching condition depends on plasma parameters, thus capacitors in the matching circuit are adjusted to minimize the reflected power. Typical plasma parameters, at the conditions of 900 G magnetic field strength and 5 mTorr argon gas pressure, are ~6 × 10^18 ^m^−3^ of center plasma density and ~2.5 eV electron temperature. The LMD-U device was shutdown at the end of 2008. The upgrade device of the LMD-U is the PANTA (Plasma Assembly for Nonlinear Turbulence Analysis)^27^.

### Reconstruction of cross-section image

The azimuthal cross-section image is reconstructed by using a 64-channel azimuthal probe array and a radially movable probe. A local peak of density bump rotates in the azimuthal direction quasi-periodically. The time at the peak of density bump in each period, *t*_0_, is detected with the azimuthal probe array at *r*_0_ = 4 cm by using the template method^28^. Then the delay time for *i*-th period, *τ*, is defined as *τ* = *t* − *t*_0_(*i*). In this manner, the azimuthal location of density bump, *θ*_*p*_(*r*_0_, *τ, i*), is also obtained from the azimuthal probe array. If the density bump is coherent, *θ*_*p*_(*r*_0_, *τ* = 0, *i*) is unchanged. However, the density bump is a quasi-periodical phenomenon and thus *θ*_*p*_(*r*_0_, *τ* = 0, *i*) varies randomly depending on the trigger. Based on the trigger time *t*_0_, ion saturation current *I*_is_(*θ*_0_, *r, τ, i*) and floating potential *V*_f_(*θ*_0_, *r, τ, i*) are obtained from a radially movable probe, where *V*_f_ = (*V*_f, up_ + *V*_f__, down_)/2 and *θ*_0_ = 0. The relative azimuthal location of the movable probe with respect to the location of the density bump, *θ_i_ *= *θ*_*p*_(*r*_0_, *τ *= 0, *i*) − *θ*_0_, is changed randomly to cover the entire region of azimuthal angle after many cycles. Thereby *θ*_*i*_ is understood as *θ*. By assembling 2015 periods of signals, we can obtain a data set of *I*_is_(*θ, r, τ*) and *V*_f_(*θ, r, τ*), and reconstruct the two-dimensional structure of these perturbations, 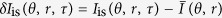 or 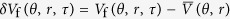, where 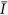 (or 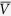) is a long-time average of *I*_is_(*θ, r, τ*) (or *V*_f_(*θ, r, τ*)). The vorticity 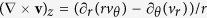 is also reconstructed from 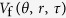, where **v** indicates the velocity vector composed of *v*_*r*_ and *v*_*θ*_ at each radial and azimuthal position. *v*_*r*_ and *v*_*θ*_ are calculated from *E*_*θ*_/*B* and *E*_*r*_/*B*, where *E*_*r*_ = 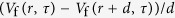, *d *= 5 mm, *E*_*θ*_ = 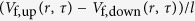, and 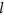 = 1 cm. Fluctuations with high azimuthal mode number (*m* > 8) at each radial position are subtracted because of large noise. The azimuthal phase velocity of solitary wave is also subtracted. By using this new method, a dynamical structural change synchronized with zonal perturbation on an azimuthal cross-section is revealed.

## Additional Information

**How to cite this article**: Arakawa, H. *et al*. Eddy, drift wave and zonal flow dynamics in a linear magnetized plasma. *Sci. Rep.* **6**, 33371; doi: 10.1038/srep33371 (2016).
